# Effect of restrictive versus liberal fluid therapy for laparoscopic gastric surgery on postoperative complications: a randomized controlled trial

**DOI:** 10.1007/s00540-024-03439-w

**Published:** 2024-12-16

**Authors:** Yusuke Kusaka, Takeshi Ueno, Toshiaki Minami

**Affiliations:** Department of Anesthesiology, Osaka Medical and Pharmaceutical University, 2-7 Daigaku-machi, Takatsuki, Osaka 569-8686 Japan

**Keywords:** Gastric surgery, Fluid therapy, Postoperative complications, Bioimpedance analysis

## Abstract

**Purpose:**

Currently, laparoscopic surgery is a standard technique in the field of abdominal surgery. However, the most adequate fluid regimen during laparoscopic surgery remains unclear. The aim of this trial is to compare a restricted fluid therapy with a liberal fluid therapy for laparoscopic abdominal surgery. Our hypothesis was that restrictive fluid therapy would reduce postoperative complications better than liberal fluid therapy.

**Method:**

In this randomized controlled trial, patients scheduled for laparoscopic gastric surgery were randomized to either the liberal group (receiving 7–10 ml/kg/h of crystalloid) or the restrictive group (receiving 1–2 ml/kg/h of crystalloid) for each stratum of surgical procedure from April 2017 to March 2019. For both groups, blood loss was replaced by an equal volume of hydroxyethyl starch. The primary endpoint was postoperative complications up to 30 days after surgery, according to the Clavien–Dindo classification.

**Results:**

We enrolled 148 patients, and 140 of these were randomized to either the liberal or the restrictive group after exclusion. As a result, 69 cases were included in the liberal group for analysis, and 67 patients composed the restrictive group. Median fluid administration for the liberal and restrictive groups was 2950 ml and 800 ml, respectively. As well, overall complications in the liberal and restrictive groups were 27.5% and 19.4%, respectively (risk ratio 0.71, 95% confidence interval 0.38–1.31, *p* value = 0.264).

**Conclusion:**

Restricted fluid therapy and liberal fluid therapy did not show any statistical differences in postoperative complications after laparoscopic gastric surgery.

**Supplementary Information:**

The online version contains supplementary material available at 10.1007/s00540-024-03439-w.

## Introduction

Anesthesiologists routinely perform fluid administration to correct preoperative dehydration, decreased preload due to the induction of anesthesia, and to replace perioperative bleeding and insensible perspiration. Although it is well known that most parts of the fluid administered during surgery end up in the extravascular spaces [1], anesthesiologists had given patients a large amount of fluid, especially in open abdominal surgery, thus resulting in a gain of body weight, changes in body composition, and occasional intestinal dysfunction [2]. The enhanced recovery after surgery (ERAS) pathway [3] has been the standard care for years, and a restrictive fluid regimen to avoid excessive water balance is recommended within it. However, a restrictive fluid regimen could increase the risk of hypoperfusion, thus causing vital organ failure [4]. A recent randomized control trial (RCT) [4] demonstrated that a restrictive fluid regimen for major abdominal surgery did not improve disability-free rate survival and was associated with a higher rate of acute kidney injury (AKI). Therefore, liberal or restrictive fluid management is both neither new nor old, and the preference for either has switched multiple times over the decades. On the other hand, currently, laparoscopic surgery has increasingly become a standard operating procedure in the field of abdominal surgery. The procedure allows surgeons to obtain a favorable visual field with small incisions and lower perioperative hemorrhage [5]. In addition, insensible evaporation from the abdominal cavity during laparoscopic surgery is thought to be less than that of open surgery. Given these advantages of laparoscopic surgery and the ERAS pathway, it would be possible to place more restrictions on the perioperative fluid volume. Nevertheless, the most adequate fluid regimen during laparoscopic surgery has not yet been established.

As mentioned above, it is well known that a large amount of fluid affects body composition, and various methods [6] can be used to assess it. Among them, the bioimpedance analysis (BIA) is simple, minimally invasive, and can be performed at the bedside. It is also used to assess body composition, including total and extracellular water volumes, based on tissues’ capacity to conduct electrical impulses [7]. BIA was previously recommended for evaluating fluid status in dialysis patients; however, its use has now been expanded to include septic patients [8], burn patients [9], and even acute high-risk abdominal surgical patients [10]. The BIA method is suitable for assessing the effect of fluid volume on changes in the body composition of postoperative patients.

From these perspectives, the authors conducted a randomized control trial to compare a restricted fluid therapy with a traditional, liberal fluid therapy for laparoscopic abdominal surgery using BIA to assess postoperative body composition. The aim of this trial was to optimize fluid volume in laparoscopic gastric surgery and to investigate the difference in body composition with intraoperative fluid volume using BIA. Our hypothesis was that restrictive fluid therapy would reduce postoperative complications better than liberal fluid therapy.

## Methods

### Population

We enrolled those patients undergoing elective laparoscopic upper gastrointestinal surgery in Osaka Medical College hospital in a single-blinded randomized clinical trial from April 2017 to March 2019. Patients were excluded if they were over 85 years old or if they had end-stage kidney failure requiring hemodialysis or a history of permanent pacemaker implantation, the reason being that bioimpedance analysis may have affected the cardioelectric device. Patients were informed by the attending anesthesiologist before surgery and gave both their oral and written consent for participation of this study. The protocol of this study was approved by the institutional ethics committee (application number: 1883, date of approval: 3/7/2016) and registered with the UMIN Clinical Trial Registry (ID UMIN000022351).

### Setting

This single-center trial was conducted in the operating room of Osaka Medical and Pharmaceutical University Hospital.

### Intervention

#### Randomization

Trial participants were stratified prior to randomization to avoid any bias in the surgical procedure for each group. Stratification was performed for 3 types of techniques: laparoscopic distal gastrectomy, laparoscopic proximal gastrectomy, and laparoscopic total gastrectomy. For each stratum, the participants were then preoperatively randomized to either the restrictive fluid group (restrictive group) or the liberal fluid group (liberal group) by the permuted block method using a random number table. Both surgeons and the patients were kept perioperatively blinded as to which fluid therapy was applied.

#### Anesthesia

General anesthesia was induced with fentanyl (4 mcg/kg) and propofol (1.5 mg/kg), and neuromuscular blocking was provided by rocuronium (0.6 mg/kg). After tracheal intubation, anesthesia was maintained with desflurane (4–5%) and remifentanil (0.1–0.5 mcg/kg/min). An additional administration of rocuronium (0.4 mg/kg) was performed under the guidance of the peripheral nerve stimulation (train-of-four monitoring). Mechanical ventilation was performed using a semiclosed circuit (Fabius, Drägel, Germany) with FiO_2_ 25% to 40%, tidal volume of 8 ml/kg, respiratory rate 10–15 breaths per minute, positive end-expiratory pressure 5 cmH_2_O, and a total gas flow of 2/L/min. After pneumoperitoneum, the respiratory rate was adjusted to keep partial pressure of carbon dioxide between 35 and 45 mmHg using arterial blood gas analysis. The patients were monitored with an electrocardiogram, and heart rate (HR), radial arterial pressure (RAP), pulse pressure variation (PPV), percutaneous oxygen saturation, end-tidal carbon dioxide tension, bispectral index, temperature, urine output, and train-of-four ratio were monitored as well. At the end of the pneumoperitoneum, fentanyl (5 mcg/kg) and acetaminophen (15 mg/kg) were administered, and an ultrasound guided rectus sheath block and bilateral transversus abdominis plane block were performed with levobupivacaine (1 mg/kg). After the administration of sugammadex (4 mg/kg) as a neuromuscular blockade reversal agent, patients were extubated, and the nasogastric tube was then removed. Postoperative analgesia was performed via a continuous infusion of fentanyl (0.5 mcg/kg/h) for 24 h under the careful and continuous monitoring of the respiratory rate and oxygen saturation. During surgery, the patient was placed in a supine position with both legs spread apart and 5 degrees from the horizontal, and the pneumoperitoneum pressure was kept at 10 mmHg.

#### Perioperative fluid management

**Fig. 1 .**
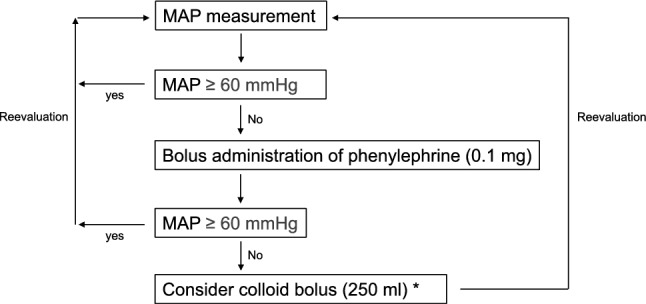
Details of fluid therapy for both groups. *MAP* mean arterial pressure

None of the patients received perioperative bowel preparation, and all were allowed to drink 250 ml of Arginaid water® (Nestlé Health Science, Vevey, Switzerland) until 2 h before general anesthesia. Details of fluid therapy for both groups are shown in Fig. 1. In both the liberal and restrictive groups, an infusion of a balanced crystalloid solution (bicarbonated Ringer’s solution, Bicarbon®, Ajinomoto Co., INC., Tokyo, Japan) at a dose of 7–10 and 1–2 ml/kg/h was administered from the induction of anesthesia to the end of surgery. For both groups, blood loss was replaced by the administration of an equal volume of colloid solution (6% hydroxyethyl starch 130/0.4, Voluven®, Fresenius Kabi, Bad Homburg, Germany), and hypotension (mean arterial pressure < 60 mmHg) was treated with 0.1 mg of phenylephrine per dose during the induction of anesthesia and surgery. If phenylephrine was not effective, or the hypotension was thought to be caused by hypovolemia (considering the patient’s HR), RAP, and PPV, 250 ml of Voluven® could be given at the discretion of the attending anesthesiologist. Blood loss was measured by the operating room nurse by summing the amount aspirated in the surgical field and the amount contained in the used gauze. All patients were administered 1 g of cefazolin as a perioperative antibiotic every 4 h during surgery.

#### Postoperative management

For both groups, postoperative management was performed according to the same clinical pathway for laparoscopic gastrectomy. Fluid infusion was continued postoperatively at a dose of 1.5 ml/kg/h until the patient could take water on the day of the surgery. Low urine output under 0.5 ml/kg/h without hypotension and hypovolemia was treated with furosemide. No interventions other than the administration of diuretics for low urine output, including additional postoperative fluid administration, were performed. Postoperative pain was treated with acetaminophen or fentanyl as needed, and nausea was treated with metoclopramide.

#### Bioimpedance analysis

Perioperative body composition measurements were performed with the Inbody720® (Inbody Co., Ltd., Seoul, Korea) that was placed in the patient ward. This device utilizes hand-to-foot bioelectrical BIA that sends varying frequencies of alternating current through the body. These impedance values are then used to predict total body water (TBW), including extracellular water (ECW) and intracellular water (ICW) [7]. The analyzer utilizes a tetrapolar 8-point tactile electrode system. The Inbody720® also takes 30 impedance measurements with 6 frequencies (1, 5, 50, 250, 500, and 1000 kHz) with a test duration of about 60 s. High-frequency currents pass through the TBW, whereas low-frequency currents cannot penetrate cell membranes and flow exclusively through the ECW. When performing measurement with this device, patient data (age, sex, and height) must be entered into the software for each patient. Before contact with the electrodes, patients cleansed their hands and feet with alcohol swabs. Standing in an upright position, the feet are then centered on the electrodes, and the hand electrodes are grasped with the arms being fixed. The patients are careful that there is no contact between the arms and the torso, and this position is held, while measurement is performed. In the study, body weight (BW), TBW, ECW, and ECW/TBW were measured every morning on postoperative day 0 (POD0), POD1, POD3, POD5, and POD7. Measurements on POD0 were performed just before surgery.

#### Intraoperative data

The following intraoperative data were recorded for both groups: duration of surgery, duration of anesthesia, duration of pneumoperitoneum, total volume of fluid administered (crystalloid and colloid), urine output, blood loss, total phenylephrine dose, lactate, and creatinine levels at the end of the anesthesia. To assess the duration and extent of exposure to low blood pressure for both groups, the authors calculated the area under the threshold (AUT) and the time weighted average of the AUT (TWA-AUT) as described previously [11]. The TWA-AUT is a calculation of the depth (in millimeters of mercury) of hypotension below the “threshold” MAP of 60 mmHg multiplied by the time spent in hypotension in minutes, thus resulting in an AUT. To better compare this value between different cases, this AUT is divided by the total duration of the monitoring as follows:

$${\text{TWA}} - {\text{AUT }} = \frac{{{\text{depth of hypotension }} \times {\text{ time spent in hypotension}}}}{{\text{total duration of the monitoring}}}.$$

For example, for an MAP of 45 mmHg that lasts for 5 min, the AUC is (60–45) $\times$ 5 = 75. If the total monitoring time is 120 min, then TWA = 75/120 = 0.625.

### Outcomes

The primary outcome measures were postoperative complications up to 30 days after surgery, limited to adverse events related to the surgical procedure and general anesthesia. Complications were evaluated by the surgeons according to the Clavien–Dindo classification (CDC) [12] and were defined as grade 2 or higher. CDC was proposed by Dindo et al. [13] to grade postoperative adverse events according to their severity. Grade 1–2 is defined as minor complications and Grade 3–5 as major complications. Details of the CDC are shown in Table 1. The secondary outcome measures were deviations from a clinical pathway, length of hospital stay, and perioperative body composition (TBW, ECW, and ECW/TBW) measured using BIA. Deviation from a clinical pathway was defined as a delayed initiation of oral feeds.

**Table 1:** Classification of surgical complications

Grade	Definition
Grade 1	Any deviation from the normal postoperative course without the need for pharmacological treatment or surgical, endoscopic, and radiational interventions. Allowed therapeutic regimens are: drugs as antiemetics, antipyretics, analgetics, diuretics, electrolytes, and physiotherapy. This grade also includes wound infections opened at the bedside
Grade 2	Requiring pharmacological treatment with drugs other than those allowed for grade 1 complications. Blood transfusions and total parenteral nutrition are also included
Grade 3	Requiring surgical, endoscopic, or radiological intervention
Grade 3a	Intervention not under general anesthesia
Grade 3b	Intervention under general anesthesia
Grade 4	Life-threatening complication (including CNS complications)^a^ requiring IC/ICU management
Grade 4a	Single organ dysfunction (including dialysis)
Grade 4b	Multiorgan dysfunction
Grade 5	Death of a patient

*CNS* central nervous system, *IC* intermediate care, *ICU* intensive care unit

^a^Brain hemorrhage, ischemic stroke, subarachnoidal bleeding, excluding transient ischemic attacks

**Table 2:** Patient characteristics

	Liberal group (*n* = 69)	Restrictive group (*n* = 67)
Age	69 (64–75)	70 (62–77)
Gender (female/male)	27/42	22/45
Height (cm)	161 (155–166)	158 (156–167)
Weight (kg)	56 (52–66)	58 (49–66)
BMI (kg/cm^2^)	22 (20–25)	23 (20–24)
ASA-PS (1/2/3)	11/50/8	15/46/6
Serum creatinine (mg/dl)	0.79 (0.67–0.92)	0.85 (0.69–0.98)
Clinical stage (stage I/II–IV)	49/19	47/19
Type of surgery (LADG/LAPG/LATG)	54/7/8	52/8/7

Data are presented as median (interquartile range)

*BMI* body mass index, *ASA-PS* American Society of Anesthesiologists Physical Status, *LADG* laparoscopic distal gastrectomy, *LAPG* laparoscopic proximal gastrectomy, *LATG* laparoscopic total gastrectomy

### Statistical analysis

The sample size was calculated as follows: the frequency of postoperative complications (Clavien–Dindo grade 2 or higher) was assumed to be 20% overall [14, 15], 30% in the liberal group, and 10% in the restricted group. The number of patients included in this trial was then calculated to be 126 at a significance level of 0.05 and a power of 0.80. The number was increased to 140 considering that withdrawals and failures to follow-up occur in about 10% of cases. The statistical analysis was performed by the modified intention-to-treat principle. Cases in which the surgery was not performed or in which the patient had to be transferred to open surgery were not included in the analysis. As for patient background and perioperative data, continuous variables were presented as a median with an interquartile range and analyzed by the Mann–Whitney *U* test. Frequencies were presented as a number or a percentage and analyzed by the *χ*^2^ test. The primary outcome of postoperative complications according to the CDC was analyzed by calculating the relative risk ratio, and the secondary outcome of perioperative body composition was analyzed by the linear mixed effect model to assess chronological transition of outcome variables within each group and between both groups. Groups were treated as fixed effects, and each patient was treated as a random effect. In addition, for between-group analysis, the authors incorporated the interaction term between groups and measurement time (POD0, 1, 3, 5, and 7) into the model to assess the effect modification. Toeplitz was used as a covariance structure of repeated measures, and the Dunnett correction was used for the multiple comparison test. A *p* value < 0.05 was considered statistically significant, and < 0.0125 was considered significant for a multiple comparison test. Toeplitz is one of the covariance structures often used in the analysis of repeated measured data using mixed effect models. Toeplitz is characterized by the fact that the variance of the data at each time point is equal, and the correlations at the same interval (e.g., POD1 and POD3, POD3, and POD5) are all equal as well.

## Results

**Fig. 2:**
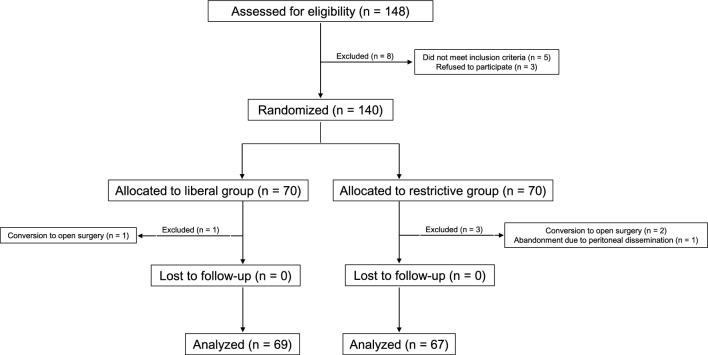
Patient flow chart

**Fig. 3:**
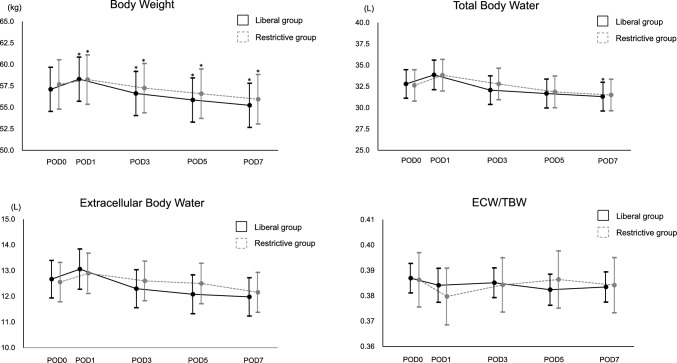
Chronological transition of body composition. Data are presented as mean with a 95% confidence interval. No significant differences were seen between both groups. *p < 0.0125 compared with POD0. *BW* body weight, *TBW* total body water, *ECW* extracellular body water

A patient flowchart is shown in Fig. 2. From April 2017 to March 2019, a total of 148 patients met the eligibility requirements, five did not meet the inclusion criteria, and three refused to participate in the trial. As a result, we randomized 140 patients to the restrictive group (70 patients) and to the liberal group (70 patients), and one patient from the restrictive group was excluded from the analysis due to the conversion to open surgery. As well, three patients from the liberal group were excluded from the analysis as the result of the perioperative conversion to open surgery (2 patients) and the interruption of operation (one patient) after peritoneal dissemination was discovered. The patient background at baseline and preoperative comorbidities are shown in Table 2 and Supplementary Table 1, respectively. Table 3 shows the perioperative data, and no statistically significant differences were seen between the two groups except for urine output and postoperative serum creatinine levels obtained from the blood exam immediately after surgery. According to these creatinine levels, six patients (8.7%) in the restricted group had AKI, five of whom met the criteria of stage 1, and one met the criteria for stage 2; on the other hand, only one patient (1.5%) in the liberal group met the criteria for AKI stage 1, which was not statistically significant (*p* = 0.116) between the two groups. The mean duration from the end of surgery to oral fluid intake was 21 h 15 min for the R group and 20 h 59 min for the L group. Tables 4 and 5 show the postoperative course and complications. Overall complications for the liberal and restrictive groups were 27.5% and 19.4%, respectively (risk ratio 0.71, 95% confidence interval 0.38–1.31, *p* value = 0.264). Two patients in the liberal group were admitted to the intensive care unit (ICU) for mechanical ventilation to treat aspiration pneumonia and for the postoperative management of intraabdominal hemorrhage due to pancreatic fistula. Two patients in the liberal group were readmitted for the emergent operation of Petersen’s hernia and for the antibiotic treatment of cervical abscess. Two patients in the restrictive group were readmitted for antibiotic treatment of liver abscess and for endoscopic hemostasis to gastrointestinal hemorrhage requiring ICU admission. Another patient in the restrictive group received endoscopic hemostasis to gastrointestinal hemorrhage requiring ICU admission as well. Moreover, no significant differences between the two groups were seen as for deviations from the clinical pathway, length of hospital stay, and readmission. Details of the overall postoperative complications are shown in Supplementary Table 2. Complications that were particularly associated with excessive fluid volume, such as anastomotic leakage, ileus, surgical site infection, and pleural effusion, occurred in 10.1% of patients in the liberal group and 9.0% in the restrictive group. Perioperative chronological changes of BW, TBW, ECW, and ECW/TBW are shown in Fig. 3. As for the within-group analysis, BW of both groups increased significantly on POD 1 and decreased significantly on POD 3, 5, and 7, compared that on POD 0. Similarly, TBW and ECW for both groups increased on POD 1 and decreased subsequently on POD 3, 5, and 7, not significantly compared with those on POD 0. As for the between-group analysis of BW, the group was not statistically significant (*p* value = 0.789); however, the interaction term (group × day) was statistically significant covariate (*p* value = 0.002). As for TBW, ECW, and ECW/TBW, neither group nor interaction term was statistically significant. The results of the intergroup multiple comparison of body composition are shown in Supplementary Table 3.

**Table 3:** Intraoperative data

	Liberal group (*n* = 69)	Restrictive group (*n* = 67)	*p* value
Duration of surgery (min)	252 (203–309)	250 (211–297)	0.924
Duration of anesthesia (min)	333 (285–393)	333 (296–376)	0.929
Duration of pneumoperitoneum (min)	214 (180–260)	220 (186–261)	0.665
Fluid administration (crystalloid) (ml)	2950 (2400–3650)	400 (270–500)	NA
Fluid administration (colloid) (ml)	0 (0–0)	400 (200–600)	NA
Total fluid administration (ml)	2950 (2400–3650)	800 (650–950)	NA
Urine output (ml)	170 (115–335)	120 (70–225)	0.001
Blood loss (ml)	10 (5–20)	5 (5–20)	0.159
Phenylephrine (mg)	0.9 (0.5–2.1)	1 (0.55–1.9)	0.655
TWA-AUT (mmHg)	0.86 (0.31–1.53)	0.95 (0.41–1.52)	0.236
PaO_2_/FiO_2_ ratio	392 (325–431)	411 (327–502)	0.069
Lactate (mg/dl)	12.1 (9.5–14.5)	10.3 (8.8–14)	0.204
Postoperative serum creatinine (mg/dl)	0.86 (0.67–1.00)	1.06 (0.76–1.28)	0.002

Data are presented as median (interquartile range)

*TWA-AUT* time weighted average area under the threshold, *NA* denotes not applicable

**Table 4:** Primary outcome

Complications	Liberal group	(*n* = 69)	Restrictive group	(*n* = 67)	RR	(95%CI)	*p* value
Overall	19	(27.5%)	13	(19.4%)	0.71	(0.38–1.31)	0.264
CD grade 2	14	(20.3%)	9	(13.4%)	–	–	
CD grade 3	3	(4.3%)	4	(6.0%)	–	–	
CD grade 4	2	(2.9%)	0	(0.0%)	–	–	

*RR* risk ratio, *CI* confidence interval, *CD* Clavien–Dindo classification

**Table 5:** Secondary outcome

	Liberal group (*n* = 69)		Restrictive group (*n* = 67)		RR		(95%CI)			*p* value
Deviation from clinical pathway	11 (15.9%)		15 (15.9%)		1.40		(0.70-2.83)			0.339
Length of hospital stay (day)	11 (10–13)		11 (10–13)		NA					0.995

*RR* risk ratio, *NA* denotes not applicable

## Discussion

This is a randomized control trial involving only laparoscopic gastric surgical patients to evaluate the effect of perioperative fluid administration on postoperative complications.

The ERAS pathway is known to contribute to early postoperative recovery [16] and improved short-term prognosis in gastric surgical patients [14]. It is well known that the ERAS pathway for upper gastrointestinal surgery recommends restriction of intraoperative fluid administration [17]. However, a retrospective observational study of gastric surgical patients reported that the adherence rate for intraoperative fluid restriction was relatively low [18]. Therefore, from this perspective, this clinical trial is considered novel and meaningful. While there have been many studies examining intraoperative fluid therapy, only a few studies [19–21] have limited the target population to solely laparoscopic surgical patients. Our trial could not demonstrate that restrictive fluid therapy reduces postoperative complications better than liberal fluid therapy. Since Brandstrup et al. first reported in 2003 that a restrictive fluid regimen reduced complications after elective colorectal surgery, several RCTs have been conducted to verify the effect of perioperative restrictive or liberal approaches on postoperative complications and mortality. Recently, an unprecedentedly large RCT [4] involving a total of 2,983 patients was conducted by Myles et al., and it showed that the disability-free survival at 1 year did not change in patients randomized to a restricted or liberal approach, with the former strategy being related to higher rates of AKI, surgical site infection, and the need for renal-replacement therapy. Furthermore, a recent meta-analysis [22] performed by Antonio Messina et al. demonstrated that the choice between liberal or restrictive approaches did not affect overall major postoperative complications or mortality in major abdominal elective surgeries. As similar to their study, we found no statistical difference in the frequency of postoperative complications between the two fluid regimens in our study of patients who underwent laparoscopic gastrectomy. Notably, in a subgroup analysis, Messina et al. reported that a liberal fluid policy was associated with lower overall major renal complications, as compared with a restrictive policy. A restrictive fluid approach aimed to achieve a zero-balance may involve the administration of colloids, such as hydroxyethylstarch (HES) [15], as our trial did also. In our study, there was no statistically significant difference in the incidence of postoperative acute renal failure between the two groups; however, the creatinine level immediately after surgery was significantly higher in the fluid restriction group. It is unclear whether these higher rates of postoperative renal complications in the restricted fluid approach are due to HES administration or renal hypoperfusion. However, considering that a recent, large RCT [23] comparing the intraoperative administration of HES versus saline for intravascular volume replacement in high-risk surgical patients found no significant difference in a composite outcome of death or major postoperative complications, intraoperative administration of HES for a restrictive fluid approach would be acceptable. Nevertheless, fluid restriction is not without its benefits. According to a systematic review of infusion studies [24] in laparoscopic surgical patients, goal-directed fluid therapy was found to accelerate gastrointestinal recovery and shorten the length of hospital stay. This review also showed that goal-directed fluid therapy resulted in lower total volume of infusions. These results suggest that “appropriate” infusion restrictions could ameliorate outcomes for patients undergoing laparoscopic surgery. Although it is difficult to perform appropriate fluid management under limited monitoring, pulse pressure variation guided fluid management has been reported to be useful in kidney transplantation [25].

Another characteristic of this trial was that we assessed postoperative body composition using BIA. Although all parameters (BW, TBW, and ECW) showed similar chronological transition, TBW and EBW did not change significantly along with the postoperative course. Besides, as for the between-group analysis, the interaction term (group × day) was significant for BW, but not significant for TBW and EBW. In this analysis, a significant interaction effect indicates that each group has a slope with a statistically different angle. These results obtained from BIA demonstrated that fluid therapy did not cause major differences in body composition, despite the large disparity in the median volume (2,950 ml in the liberal group vs. 800 ml in the restrictive group) of perioperative fluid administration between the two groups in our trial. Moreover, we assessed the ECW/TBW ratio that normally ranges from 0.36 to 0.4, with values above 0.4 indicating edematous status [26]. In this study, the ECW/TBW ratio was generally maintained below 0.4, and no significant differences were seen between the two groups. This fact suggests that there was no difference in the degree of edema between the two groups, regardless of the amount of infusion in both the R and L groups. Over the past several decades, BIA has been increasingly applied for the diagnosis of metabolic diseases, such as obesity and sarcopenia [27], as well as establishing dry weight in hemodialysis [28, 29] and fluid management in heart failure patients [30]. In recent years, a few studies reported the usefulness of BIA in perioperative fluid management and the assessment of volume status. Adi-lonut et al. concluded that absolute fluid overload (AFO), that is the difference between expected ECW and the actual measured ECW, was significantly correlated with ICU length of stay in their prospective observational study of patients scheduled for major open abdominal surgery [31]. Moreover, Cihoric et al. demonstrated that perioperative overhydration measured with BIA was associated with worse outcomes, compared with patients with normohydration or dehydration, in their prospective observational study of patients undergoing acute high-risk abdominal surgery [10]. According to these studies, BIA-assessed fluid status might be a helpful measure for the management of the patients after major invasive surgery.

This study has some limitations. First, with respect to the primary outcome, a difference of 8% was obtained between the two groups. Although this nearly 10% difference is not negligible from a clinical point of view, the lack of statistical significance could be attributed to a low power due to the small sample size of this trial. Second, in this study, the overall median fluid administration in the liberal group was 2,950 ml. In a recent meta-analysis [22], Messina et al. stratified their studies according to three tertiles of the overall amount of perioperative fluid given at day 0 and day 1. In the lowest tertile, fluid amount was under 3,673 ml, and 10 of 12 studies were included within the restrictive group. Although using this stratification, our liberal group had the possibility of being classified into the restrictive group. However, considering that our accumulated data represent only those infusion volumes administered intraoperatively (day 0), a median infusion volume of 2,950 ml seems to be appropriate as a liberal fluid regimen. Third, although BIA is a non-invasive and simple body composition analyzing technique, it should be kept in mind that the body composition values indicated by BIA are estimates, not measurements. Especially, in critically ill or obese patients, the reliability of these parameters decreases.

In conclusion, restricted fluid therapy and liberal fluid therapy did not show any statistical differences in postoperative complications (Clavien–Dindo grade 2 or higher) after laparoscopic gastric surgery.

## Supplementary Information

Below is the link to the electronic supplementary material.

Supplementary file1 (DOCX 25 KB)

## Data Availability

Data are available upon resonable request.
