# Supplementary material for: Effect of restrictive versus liberal fluid therapy for laparoscopic gastric surgery on postoperative complications: a randomized controlled trial
Source: J Anesth. 2024 Dec 16;39(1):101–10. doi: 10.1007/s00540-024-03439-w (PMC11782308; doi:10.1007/s00540-024-03439-w)
Supplement: Supplementary file 1 — Supplementary file1 (DOCX 25 KB) [file 540_2024_3439_MOESM1_ESM.docx]

| Supplementary table 1 Preoperative comorbidities | | |
| --- | --- | --- |
| Preoperative comorbidities | Liberal group (n = 69) | Restrictive group (n = 67) |
| Hypertention | 37 | 31 |
| Diabetes mellitus | 12 | 16 |
| Hyperlipidemia | 10 | 12 |
| Obesity | 3 | 0 |
| Ischemic heart disease | 0 | 6 |
| Valuvlar heart disease | 1 | 2 |
| ECG abnormality | 5 | 6 |
| Cardiomyopathy | 0 | 1 |
| Peripheral artery disease | 0 | 4 |
| Deep vein thrombosis | 4 | 2 |
| History of pulmonary embolism | 2 | 0 |
| Chronic aortic dissection | 0 | 1 |
| History of smoking | 10 | 7 |
| Current smoker | 1 | 1 |
| Chronic obstructive pulmonary disease | 6 | 3 |
| Interstitial pneumonia | 2 | 0 |
| Asthma | 1 | 0 |
| History of lung resection | 1 | 2 |
| Sleep apnea syndrome | 0 | 1 |
| Chronic renal failure | 1 | 5 |
| End stage renal failure | 0 | 0 |
| Preoperative chemotherapy | 5 | 6 |
| Chemotherapy for other cancer | 0 | 1 |
| Post endoscopic submucosal dissection | 2 | 2 |
| Elevated transaminase levels | 0 | 1 |
| History of subarachnoid hemorrhage | 1 | 1 |
| History of cerebral infarction | 4 | 2 |
| Stenosis of internal carotid artery | 0 | 1 |
| Collagen diiease | 2 | 3 |
| Dimentia | 0 | 4 |
| Schzophrenia | 0 | 1 |

Supplementary table 2 Postoperative complications

| Complications | Diagnostic criteria | Liberal group (n = 69) | Restricted group (n = 67) |
| --- | --- | --- | --- |
| Anastomotic leakage | Symptomatic leakage needing antibiotic treatment | 2 | 1 |
|  |  |  |  |
| Ileus | Clinical findings and needing insertion of nasogastric tube | 3 | 4 |
|  |  |  |  |
| Pancreatic fistula | Needing reoperation or insertion of intraabdominal drainage tube* | 1 | 1 |
|  | Needing antibiotic treatment | 0 | 2 |
|  |  |  |  |
| Petersen's hernia | Needing reoperation* | 1 | 0 |
|  |  |  |  |
| Chylous ascites | Needing insertion of intraabdominal drainage tube* | 1 | 0 |
|  |  |  |  |
| Intraabdominal hemorrhage | Needing surgical hemostasis* | 1 | 0 |
|  |  |  |  |
| Gastrointestinal hemorrhage | Needing endoscopic hemostasis* | 0 | 2 |
|  |  |  |  |
| Surgical site infection | Needing antibiotic treatment without surgical debridment | 1 | 1 |
|  |  |  |  |
| Intraabdominal abcess | Needing insertion of intraabdominal drainage tube* | 0 | 1 |
|  | Needing antibiotic treatment | 2 | 0 |
|  |  |  |  |
| Pyothorax | Needing insertion of chest drainage tube* | 0 | 1 |
|  |  |  |  |
| Aspiration pneumonia | Needing mechanical ventilation* | 1 | 0 |
|  | Needing antibiotic treatment or oxygen therapy | 4 | 4 |
|  |  |  |  |
| Pleural effusion | Neeeding insertion of chest drainage tube* | 1 | 0 |
|  | Requiring medical treatment | 1 | 4 |
|  |  |  |  |
| Atelectasis | Verified by chest X-ray | 1 | 0 |
|  |  |  |  |
| Liver abcess | Verified by CT scan and needing antibiotic treatment | 0 | 1 |
|  |  |  |  |
| Cervical abscess | Verified by CT scan and needing antibiotic treatment | 1 | 0 |
|  |  |  |  |
| Delirium | Requiring medical treatment | 2 | 0 |
|  |  |  |  |
| Hyperglycemia | Requiring medical treatment | 1 | 0 |
|  |  |  |  |
| Urinary tract infection | Requiring antibiotic treatment | 2 | 1 |

* Major complication.

Supplementary table 3 Intergroup multiple comparison of body composition.

|  | BW | | | | TBW | | ECW | | ECW/TBW | |
| --- | --- | --- | --- | --- | --- | --- | --- | --- | --- | --- |
| Group | Liberal | | Restrictive | | Liberal | Restrictive | Liberal | Restrictive | Liberal | Restrictive |
| POD0 vs. POD1 | < | 0.001 | < | 0.001 | 0.082 | 0.014 | 0.255 | 0.337 | 0.509 | 0.420 |
| POD0 vs. POD3 |  | 0.001 |  | 0.002 | 0.194 | 0.720 | 0.242 | 0.894 | 0.644 | 0.802 |
| POD0 vs. POD5 | < | 0.001 | < | 0.001 | 0.051 | 0.119 | 0.072 | 0.881 | 0.256 | 0.986 |
| POD0 vs. POD7 | < | 0.001 | < | 0.001 | 0.009 | 0.020 | 0.032 | 0.257 | 0.376 | 0.793 |

BW, body weight; TBW, total body water; ECW, extracellular body water; POD, postoperative day.

P < 0.0125 was considered statistically significant.
